# Fusogenic Liposomes Increase the Antimicrobial Activity of Vancomycin Against *Staphylococcus aureus* Biofilm

**DOI:** 10.3389/fphar.2019.01401

**Published:** 2019-11-29

**Authors:** Andreia Borges Scriboni, Verônica Muniz Couto, Lígia Nunes de Morais Ribeiro, Irlan Almeida Freires, Francisco Carlos Groppo, Eneida de Paula, Michelle Franz-Montan, Karina Cogo-Müller

**Affiliations:** ^1^Department of Physiological Sciences, Piracicaba Dental School, University of Campinas, Piracicaba, Brazil; ^2^Department of Biochemistry and Tissue Biology, Biology Institute, University of Campinas, Campinas, Brazil; ^3^Department of Oral Biology, University of Florida College of Dentistry, Gainesville, FL, United States; ^4^Faculty of Pharmaceutical Sciences, University of Campinas, Campinas, Brazil

**Keywords:** fusogenic liposomes, cationic liposomes, *Staphylococcus aureus*, vancomycin, biofilm

## Abstract

**Objective:** The aim of the present study was to encapsulate vancomycin in different liposomal formulations and compare the *in vitro* antimicrobial activity against *Staphylococcus aureus* biofilms.

**Methods:** Large unilamellar vesicles of conventional (LUV VAN), fusogenic (LUV_fuso_ VAN), and cationic (LUV_cat_ VAN) liposomes encapsulating VAN were characterized in terms of size, polydispersity index, zeta potential, morphology, encapsulation efficiency (%EE) and *in vitro* release kinetics. The formulations were tested for their Minimum Inhibitory Concentration (MIC) and inhibitory activity on biofilm formation and viability, using methicillin-susceptible *S. aureus* ATCC 29213 and methicillin-resistant *S. aureus* ATCC 43300 strains.

**Key Findings:** LUV VAN showed better %EE (32.5%) and sustained release than LUV_fuso_ VAN, LUV_cat_ VAN, and free VAN. The formulations were stable over 180 days at 4°C, except for LUV VAN, which was stable up to 120 days. The MIC values for liposomal formulations and free VAN ranged from 0.78 to 1.56 µg/ml against both tested strains, with no difference in the inhibition of biofilm formation as compared to free VAN. However, when treating mature biofilm, encapsulated LUV_fuso_ VAN increased the antimicrobial efficacy as compared to the other liposomal formulations and to free VAN, demonstrating a better ability to penetrate the biofilm.

**Conclusion:** Vancomycin encapsulated in fusogenic liposomes demonstrated enhanced antimicrobial activity against mature *S. aureus* biofilms.

## Introduction

*Staphylococcus aureus* (*S. aureus*) is a Gram-positive microorganism responsible for the majority of nosocomial and community-acquired infections. Notably, *S. aureus* infections remain a global public health issue highly costly for the healthcare system, with increasing morbidity and mortality rates worldwide (Chakraborty et al., 2012; Honary et al., 2013; Elkhodairy et al., 2014; Holland et al., 2014). Today, over 90% of *S. aureus* strains are found to be resistant to methicillin (methicillin resistant *S. aureus*—MRSA), penicillin, aminoglycosides, macrolides, lincosamides, and other beta-lactams (Chakraborty et al., 2012; Muppidi et al., 2012; Sande et al., 2012; Elkhodairy et al., 2014; Shi et al., 2014).

In this scenario of microbial resistance, vancomycin (VAN) is considered a first-choice antibiotic for the treatment of methicillin-resistant *S. aureus* (MRSA) infections (Pumerantz et al., 2011; Honary et al., 2013; Holland et al., 2014; Men et al., 2016; Gudiol et al., 2017). While VAN remains a first-choice antibiotic for the treatment of MRSA infections, its therapeutic efficacy is limited due to its high molecular weight (1,449.2 g mol^−1^) and hydrophilicity restricting the drug interaction with bacterial cells and hindering its penetration into biofilms (Howden et al., 2010; Nicolosi et al., 2010; Butler et al., 2014; Moghadas-Sharif et al., 2015). In addition to that, VAN systemic side effects are another limiting factor, which include severe watery diarrhea, kidney failure (Pumerantz et al., 2011; Rose et al., 2012; Honary et al., 2013), ototoxicity, neutropenia, fever, anaphylaxis, thrombocytopenia, and phlebitis (McAuley, 2012).

Bacterial biofilms are characterized by the aggregation of specific bacterial species adhered to a substrate, forming highly organized microbial communities (Khameneh et al., 2014; McCarthy et al., 2015). Biofilm-forming bacteria display a differentiated phenotype compared to planktonic cells and have the ability to produce an extracellular polymeric matrix composed mainly of polysaccharides (Khameneh et al., 2014; Dong et al., 2015; McCarthy et al., 2015). This scaffold provides an extremely robust defense mechanism, which hinders antibiotic penetration into the biofilm structure, substantially reducing the susceptibility of bacterial cells to exogenous agents (Dong et al., 2015; McCarthy et al., 2015; Moghadas-Sharif et al., 2015).

The shortcomings of VAN traditional treatment along with the increased microbial resistance rates, and difficulty to treat biofilms have encouraged the development of drug-carrier systems such as VAN-loaded liposomal formulations (Kadry et al., 2004; Drulis-Kawa et al., 2009; Nicolosi et al., 2010; Lankalapalli et al., 2015). It has been shown that the liposomal sustained release of VAN (i) enhances antibacterial efficacy due to higher interaction of the antibiotic molecule with bacterial cells (Kim and Jones, 2004); (ii) improves pharmacokinetics (Ma et al., 2011); (iii) reduces toxicity (Sande et al., 2012); and (iv) increases the antimicrobial spectrum of action against Gram-negative bacteria (Nicolosi et al., 2010). Furthermore, liposomes can facilitate antibiotic penetration into bacterial cells and, therefore, increase drug concentration in the biofilm inner layers (Moghadas-Sharif et al., 2015). Despite these reports, only a few studies have evaluated the effects of liposomal formulations on the inhibition of biofilm development and viability, particularly *Staphylococcus* biofilms (Ma et al., 2011; Moghadas-Sharif et al., 2015).

The liposome composition can be specifically modulated in terms of morphology to favor the adsorption onto, or fusion with, the microbial cell membrane. Likewise, vesicle surfaces can be changed based on the characteristics of the infectious agent (Nicolosi et al., 2010). Among some types of liposomes with the ability of interacting with bacterial biofilm cells are fusogenic and cationic liposomes (Kim et al., 1999; Nicolosi et al., 2010). Fusogenic liposomes are vesicles that may fuse with biological membranes, thereby increasing drug contact and delivery into cells. They consist of lipids, such as dioleoyl-phosphatidylethanolamine (DOPE) and cholesterol hemisuccinate (CHEMS), which provide increased fluidity to the lipid bilayer and may destabilize biological membranes (Nicolosi et al., 2010; Aoki et al., 2015; Nicolosi et al., 2015). Because of their composition, fusogenic liposomes are normally in the liquid crystalline phase and, under specific chemical conditions, e.g., acidic milieu or in the presence of cations (Forier et al., 2014) they can lose the bilayer arrangement and fuse. Cationic liposomes are composed of lipids with a positive residual charge, such as stearylamine (SA), dimethyldioctadecylammonium bromide (DDBA), dimethylaminoethane carbamoyl cholesterol (DC-chol), and dioleoyltrimethylammoniumpropane (DOTAP), which provides specific electrostatic interaction with bacterial cell wall and biofilms, both negatively charged (Kim et al., 1999; Torchilin, 2012; Zhang et al., 2014; Moghadas-Sharif et al., 2015).

While fusogenic and cationic liposomes have proven advantages in interacting with bacterial cells and formed biofilms, there is still no consensus on the ideal composition of liposome-encapsulated VAN formulations able to prolong drug release and increase its antimicrobial efficacy. Thus, in the present study we developed and characterized large unilamellar vesicles of conventional (LUV VAN), fusogenic (LUV_fuso_ VAN), and cationic (LUV_cat_ VAN) liposomes encapsulating vancomycin hydrochloride. The *in vitro* antimicrobial activity of these formulations on *S. aureus* biofilms was further determined and compared.

## Materials and Methods

### Materials

VAN hydrochloride was kindly provided by Teuto/Pfizer Laboratory (Anápolis, GO, Brazil). HEPES buffer, cholesterol (Chol), alpha-tocopherol (α-T) and egg phosphatidylcholine (EPC) were purchased from Sigma-Aldrich (St. Louis, MO, USA) and chloroform was obtained from Merck (Darmstadt, Germany). Dioleoylphosphatidylethanolamine (DOPE), dipalmitoylphosphatidylcholine (DPPC), cholesterol hemisuccinate (CHEMS) and stearylamine (Sa) were purchased from Avanti Polar Lipids Inc. (Alabaster, AL USA).

### Preparation of Liposomal Formulations

Conventional (LUV VAN), fusogenic (LUV_fuso_ VAN) and cationic (LUV_cat_ VAN) liposomal formulations were prepared containing 10 mg/ml VAN. Plain, VAN-free formulations were used as negative controls in the experiments (LUV, LUV_fuso_, and LUV_cat_). All liposomal formulations were prepared with 10 mM lipid concentration, with the following composition: LUV–EPC : Chol:α-T (4:3:0.07, mol%) (Cereda et al., 2006); LUV_fuso_–DOPE : DPPC : CHEMS:α-T (4:2:4:0.07, mol%) (Nicolosi et al., 2010); LUV_cat_–EPC : Sa:Chol:α-T (1:0.5:0.5:0.07, mol%) (Kadry et al., 2004), respectively. All formulations were prepared in HEPES buffer (80 mM) containing 150 mM NaCl (pH 7.4).

Preparation of liposomal formulations was carried out as previously described, with modifications (Cereda et al., 2006). Briefly, the lipids were dissolved in chloroform, evaporated under nitrogen flow to obtain the lipid film, and vacuumed for 2 h to ensure complete solvent removal. Subsequently, the film was hydrated in HEPES buffer with or without VAN hydrochloride solution. Then the suspension was vortexed for 5 min to form large multilamellar vesicles (MLVs). The suspensions were extruded under nitrogen flow at high pressure (Extruder Emulsiflex C5, Avestin, Inc., Ottawa, ON, Canada) 12 times using polycarbonate membrane initially with 400 nm pores, and then, with 100 nm pores, to obtain small unilamellar vesicles. The extrusion of LUV_fuso_ formulation was performed in water bath at 50°C, which is higher than the DPPC phase transition temperature (Nicolosi et al., 2010).

### Characterization of Liposomal Formulations

#### Morphological Analysis

The morphology of the different types of VAN-containing liposomes or plain liposomes was analyzed by Transmission Electron Microscopy (TEM) (906 LEO-ZEISS, Jena, Germany) at 80 kV. Briefly, one drop of each formulation was added to a copper-coated grid with 200 mesh for 10 s (Electron Microscopy Sciences, Fort Washington, PA). Subsequently, uranyl acetate aqueous solution (2%, w/v) was added and kept at room temperature for 4 h.

#### Determination of Size, Polydispersity Index, Zeta Potential and Stability of Liposomes

Liposomal vesicles were diluted in deionized water for evaluation of the average size (nm), polydispersity index (PDI), and zeta potential (mV) by the dynamic light scattering using Nano ZS equipment (Malvern Instruments Ltd., Worcestershire, UK, England) at 25°C in triplicate. To evaluate stability of liposomes, these parameters were monitored during 180 days at 4°C.

#### Vancomycin Encapsulation Efficiency

The encapsulation efficiency (%EE) of VAN into liposomal formulations was determined by the ultrafiltration–centrifugation method (Da Silva et al., 2016) (35). Unencapsulated VAN was separated from encapsulated VAN by ultracentrifugation (Optima L-90K Ultracentrifuge, Beckman Coulter Inc. Pasadena, California, USA) at 120,000*g* for 2 h at 10°C. Aliquots from the supernatant were diluted in deionized water and analyzed spectrophotometrically at 280 nm (Varian Cary^®^ 50 UV-vis, Varian Inc., Palo Alto, CA, USA). The %EE was calculated based on the concentration of unencapsulated VAN over the concentration of VAN in solution, using the formula as follows:

$$\frac{\%\mathrm{EE}=[\mathrm{VAN}\;\mathrm{solution}]-[\mathrm{unencapsulated}\;\mathrm{VAN}]\times 100}{\left[\mathrm{VAN}\;\mathrm{solution}\right]}$$

#### Evaluation of Vancomycin Release *In Vitro*

The drug release assay was performed using the Franz vertical diffusion cell (Franz, 1975), which consists of two compartments—one donor and one receptor—separated by a regenerated cellulose membrane (Spectra/Por® 2) with molecular exclusion limit of 12,000–14,000 Da (Spectrum Laboratories Inc., Rancho Dominguez, CA, USA) (de Araujo et al., 2008; Da Silva et al., 2016). An aliquot of 1 ml of the liposomal suspensions was added to the donor compartment, while the receptor compartment was filled with 4 ml of buffer (pH 7.4), maintained at 37°C and 400 rpm agitation. Aliquots of the receptor medium were removed throughout the 10-hour experiment and analyzed by spectrophotometry at 280 nm (Varian Cary® 50 UV-Vis, Varian Inc., Palo Alto, CA, USA). The collected volume was replaced with fresh medium due to the dilution effect.

### Evaluation of Antimicrobial Activity

#### Microorganisms and Growth Conditions

Methicillin-susceptible *S. aureus* (MSSA) ATCC 29213 and methicillin-resistant *S. aureus* (MRSA) ATCC 43300 strains were used in this study. Microorganisms were maintained in Tryptone Soy Broth (TSB) (Difco®, New Jersey, USA) with 20% glycerol at −80°C, and cultivated onto Tryptone Soy Agar (Difco®, New Jersey, USA) plates at 37°C. Mueller Hinton Broth (MHB) (Difco®, New Jersey, USA) was used in the MIC assay, while Brain Heart Infusion (Difco®, New Jersey, USA) plus 1% D-glucose (Sigma-Aldrich, St. Louis, MO, USA) was used in the biofilm killing assays.

#### Experimental Groups

Test formulations consisted of VAN-containing and VAN-free LUV, LUV_fuso_ and LUV_cat_. The experimental groups were set as follows: A—culture medium, test formulation and inoculum; B—culture medium, control formulation and inoculum; C—culture medium, free VAN solution and inoculum; D—culture medium, HEPES buffer (vehicle) and inoculum; E—culture medium and test formulation; F—culture medium and inoculum; and G—culture medium alone.

#### Minimum Inhibitory Concentration (MIC)

The MIC was determined by the microdilution method, as previously described by the CLSI (2012), using Mueller-Hinton Broth. The formulations were added to 96-well microplates and serially diluted to obtain concentrations ranging from 0.025 to 50 µg/ml. From 18–24 h agar cultures, three to five colonies of *S. aureus* were dispersed into saline solution and bacterial inoculum was adjusted using a spectrophotometer (λ 625nm, OD 0.1, 1 to 2 × 10^8^ CFU/ml). Then, the inoculum was diluted and transferred to the wells at a final concentration of 5 × 10^4^ CFU/ml. The plates were incubated at 37°C for 24 h and the absorbance was read at 620 nm (Biochrom ASYS UVM 340, Biochrom, Cambridge, England). The MIC was defined as the lowest concentration of the formulation which inhibited visible bacterial growth. The experiments were performed in six replicates.

#### Inhibitory Effects on Biofilm Formation

The liposomal formulations were tested for their ability to inhibit biofilm formation and adherence according to the protocol proposed by Graziano et al. (2015) and Wu et al. (2013). First, BHI medium supplemented with 1% glucose and *S. aureus* cell suspension (final concentration of 5 × 10^4^ CFU/ml) were added to 96-well U-bottom microplates. Right after, the test formulations were added to the wells and plates were then incubated for 24 h, at 37°C. After this period, the supernatant was removed, and the wells were washed three times with distilled water to remove loosely bound or non-adhered cells. Biofilms were stained with 0.4% crystal violet, solubilized with 98% ethanol and read in a microplate reader at 575 nm (Asys UVM 340, Biochrom, Cambridge, England).

#### Inhibitory Effects on Biofilm Viability

The liposomal formulations were next tested for their inhibitory effects on biofilm viability, as previously described (Graziano et al., 2015) (39). Cellulose acetate membranes (25 mm diameter, 0.2 µM pores) (Sartorius Stedim GmbH, Guxhagen, Hessen, Germany) were used as substrates for *S. aureus* biofilm formation. The membranes were placed in 6-well plates containing BHI medium supplemented with 1% glucose and bacterial suspension (approximately 1 × 10^6^ CFU/ml in each well). The plates were incubated at 37°C for 24 h. Then the membranes were transferred to new plates containing fresh BHI plus 1% glucose, and biofilms were treated with the formulations at 1 × MIC, 10 × MIC, and 50 × MIC for 24 h. Treated biofilm-coated membranes were gently washed (three times) through immersion into 5 ml of 0.9% NaCl. Then, the membranes were transferred to other tubes containing freshly 5 ml of 0.9% NaCl and then sonicated with six pulses of 9.9 s, 5 s time-interval, 5% amplitude (VibraCell 400W, Sonics & Materials Inc., Newtown, CT, USA) and vortexed at 3,800 rpm for 30 s. Ten-microliter aliquots were collected from each tube, serially diluted, and plated for CFUs onto TSA. The plates were incubated at 37°C for 24 h.

### Statistical Analysis

The data distribution was analyzed using the Shapiro–Wilks test. The variables size, PDI, zeta potential, and %EE, were compared using unpaired *t*-test. Stability parameters for liposomes and the biofilm data were compared using analysis of variance (ANOVA) followed by Tukey’s post-hoc test. The drug release profile was analyzed by two-way ANOVA followed by Tukey’s post-hoc test. Statistical analyses were performed on Origin 8.0 (Microcal TM Software Inc., EUA) and GraphPad Prism 6.0 (San Diego, California, USA). The data were presented as mean and standard deviation (SD), with a 5% significance level. All data are representative of three independent experiments.

## Results

### Characterization of Liposomal Formulations

**Figure 1:**
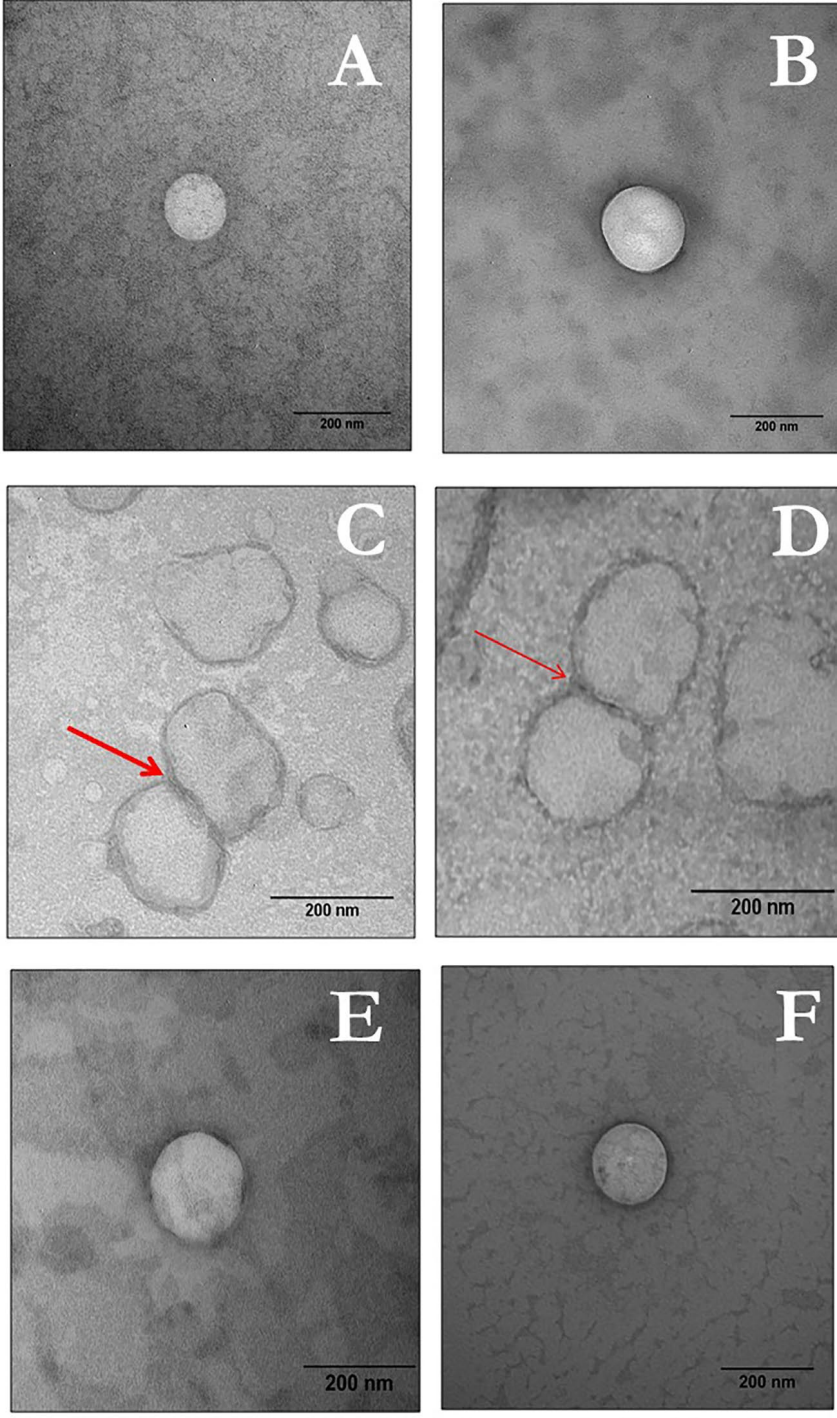
TEM images of LUV VAN, LUV_fuso_ _VAN_, and LUV_ca_ VAN.The left panel represents plain vesicles and the right panel indicated VAN-containing LUVs as follows: **(A**, **B)** LUV; **(C**, **D)** LUV_fuso_; and **(E**, **F)** LUV_cat_. Bars indicate 200 nm, with 100,000× magnification).

TEM images confirmed that the liposomal vesicles had spherical shape with clear edges. Vesicle size in all formulations ranged between 100 and 200 nm. Micrographs of all liposomal formulations are presented in **Figure 1**. As exemplified in **Figures 1C, D**, some fusogenic vesicles were found to merge with each other, which typically characterizes this type of liposome.

The means and standard deviations of size, PDI, zeta potential and %EE of the liposomal formulations are given in **Table 1**. Comparisons were made between plain and VAN-containing liposomes. No differences in size were found while PDI values increased for VAN-containing formulations in comparison to plain controls (*p* < 0.05). Moreover, as expected, the zeta potential values confirmed the presence of negative charges on LUV and LUV_fuso_ liposomes and positive charges on LUV_cat_. The encapsulation decreased the negative zeta potentials of LUV_fuso_ VAN liposomes (*p* < 0.05), while it increased the positive zeta potential in LUV_cat_ VAN, as compared to their respective controls (*p* < 0.05). Higher %EE values were observed for LUV VAN, followed by LUV_fuso_ VAN and LUV_cat_ VAN.

**Table 1:** Mean ( ± SD) of the size (nm), polydispersity index (PDI), zeta potential (mV) and encapsulation efficiency (%EE) of the liposomal formulations developed in this study.

Formulation	Size (nm ± SD)	PDI ( ± SD)	Zeta Potential (mV ± SD)	%EE ( ± SD)
**LUV**	157.53 ± 2.58	0.09 ± 0.03	−19.2 ± 5.5	–
**LUV VAN**	152.60 ± 0.80	0.17 ± 0.01*	−16.9 ± 0.5	32.5 ± 0.1
**LUV**_fuso_	161.87 ± 2.45	0.14 ± 0.02	−48.6 ± 4.9	–
**LUV**_fuso_ **VAN**	153.37 ± 0.70	0.20 ± 0.01*	−41.3 ± 2.3*	11.4 ± 0.1
**LUV**_cat_	130.97 ± 1.59	0.13 ± 0.01	50.6 ± 3.5	
**LUV**_cat_ **VAN**	139.73 ± 2.55	0.18 ± 0.02*	62.5 ± 5.6*	10.1 ± 0.1

The asterisk “*” indicates statistically significant difference between plain (LUV, LUV_FUSO,_ LUV_CAT_) and their respective vancomycin-containing liposomes (LUV VAN, LUV_FUSO_ VAN, LUV_CAT_ VAN), p < 0.05 (unpaired t-test).

**Figure 2:**
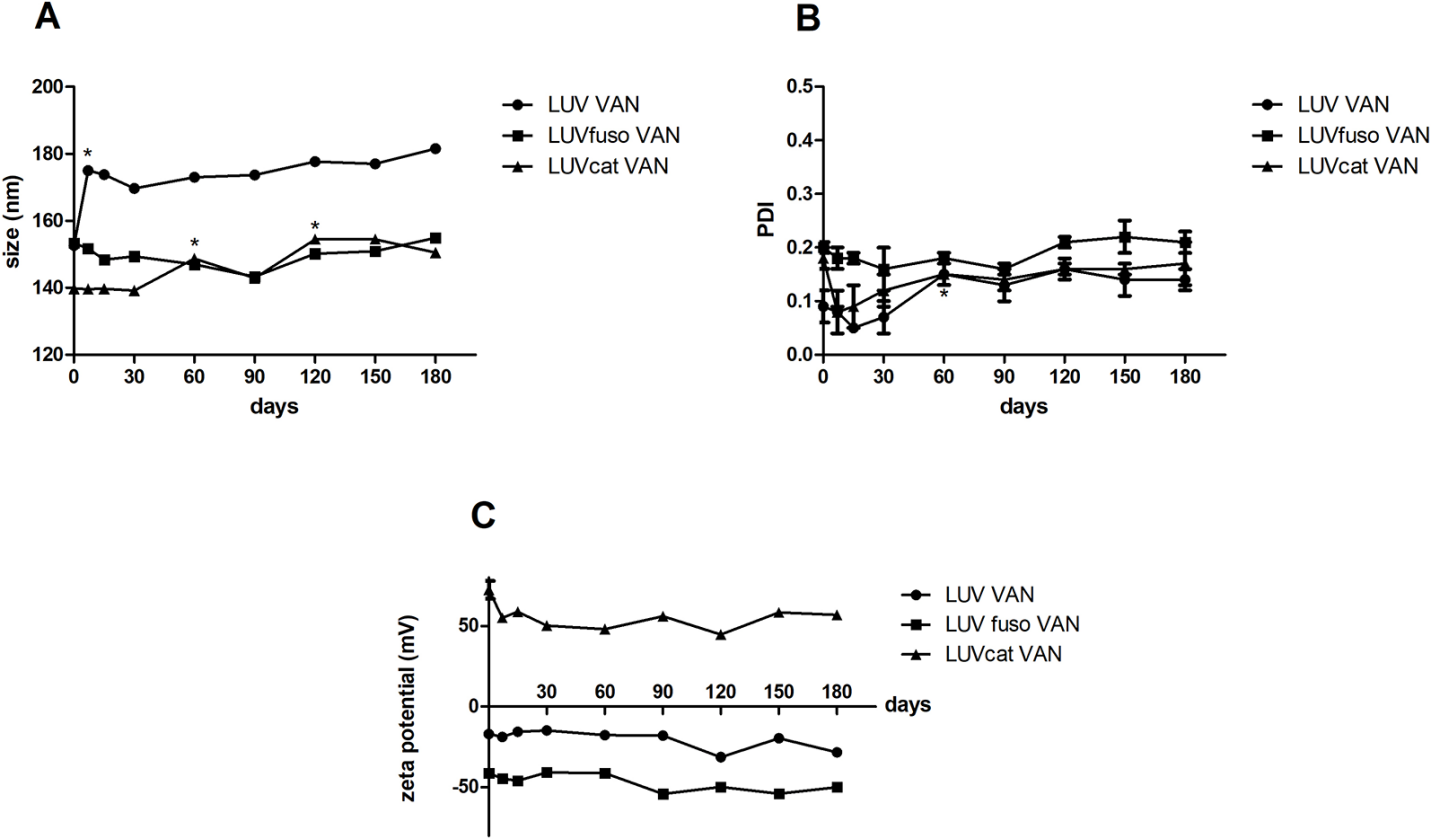
Size **(A)**, PDI **(B)** and Zeta potential **(C)** for VAN liposomal formulations analyses during 180 days. The asterisk “*” indicates statistically significant difference between the drug treatment and its respective untreated control at *p* < 0.05 (One-way ANOVA, followed by Tukey’s post-hoc test).

The stability of the formulations was determined from measurements of size, PDI and zeta potential (**Figures 2A–C**) during storage at 4°C. In general, LUV_cat_ VAN, LUV_fuso_ VAN and LUV VAN kept their size during the 180-day experimental period (p > 0.05). LUV VAN showed an increase in size after 7 days of storage (p < 0.05) but kept their size in the other time points. LUV_cat_ VAN also changed in size after 60 and 90 days (p < 0.05). However, these alterations were no greater than 10% of the initial size. No significant changes in PDI and Zeta values were found during the experiment (p > 0.05). It is also worth noting that, although with an increasingly trend, PDI values were found to be under 0.2, as required for a monodisperse size distribution.

**Figure 3:**
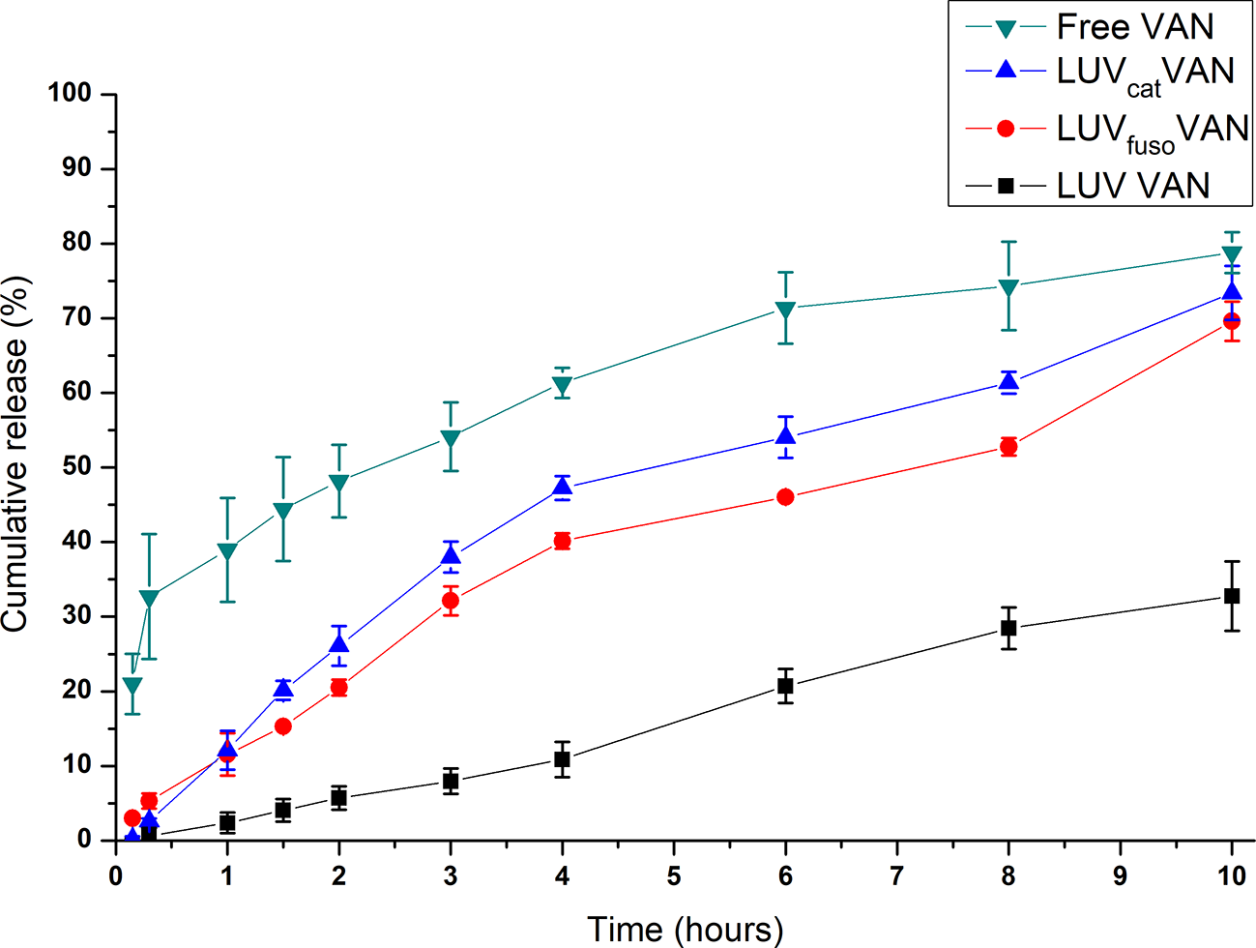
*In vitro* release kinetics of VAN in solution and encapsulated in the liposomal formulations, at 37°C. Two-way ANOVA, Tukey, P < 0.05. There were statistical difference between groups, as follows: LUV VAN × LUV_fuso_ VAN—from 1 to 10 h; LUV VAN × LUV_cat_ VAN—from 1 to 10h; LUV VAN × free VAN—from 0.15 to 10 h; LUV_fuso_ VAN × free VAN—from 0.15 to 8 h; LUVcat VAN × free VAN—from 0.15 to 8 h.

The release kinetics of VAN in solution and encapsulated in the liposomal formulations was determined *in vitro*. As seen in **Figure 3**, encapsulated VAN formulations showed prolonged releases overtime as compared to free VAN (p < 0.05).

The LUV VAN formulation showed slower release profile than the other liposomes (p < 0.05), whereas LUV_fuso_ VAN and LUV_cat_ VAN were found to have very similar release kinetics (p > 0.05). As expected, VAN-free formulations showed greater percent release at all timepoints, with a significant difference from the other liposomal formulations (p < 0.05).

### Antimicrobial Activity

Free and encapsulated VAN LUV formulations affected bacterial growth in both MSSA (29213) and MRSA (43300) strains, with MIC values ranging between 0.78 and 1.56 µg/ml. These findings are in line with the information provided by the CLSI concerning *S. aureus* susceptibility to VAN (CLSI, 2012).

**Figure 4:**
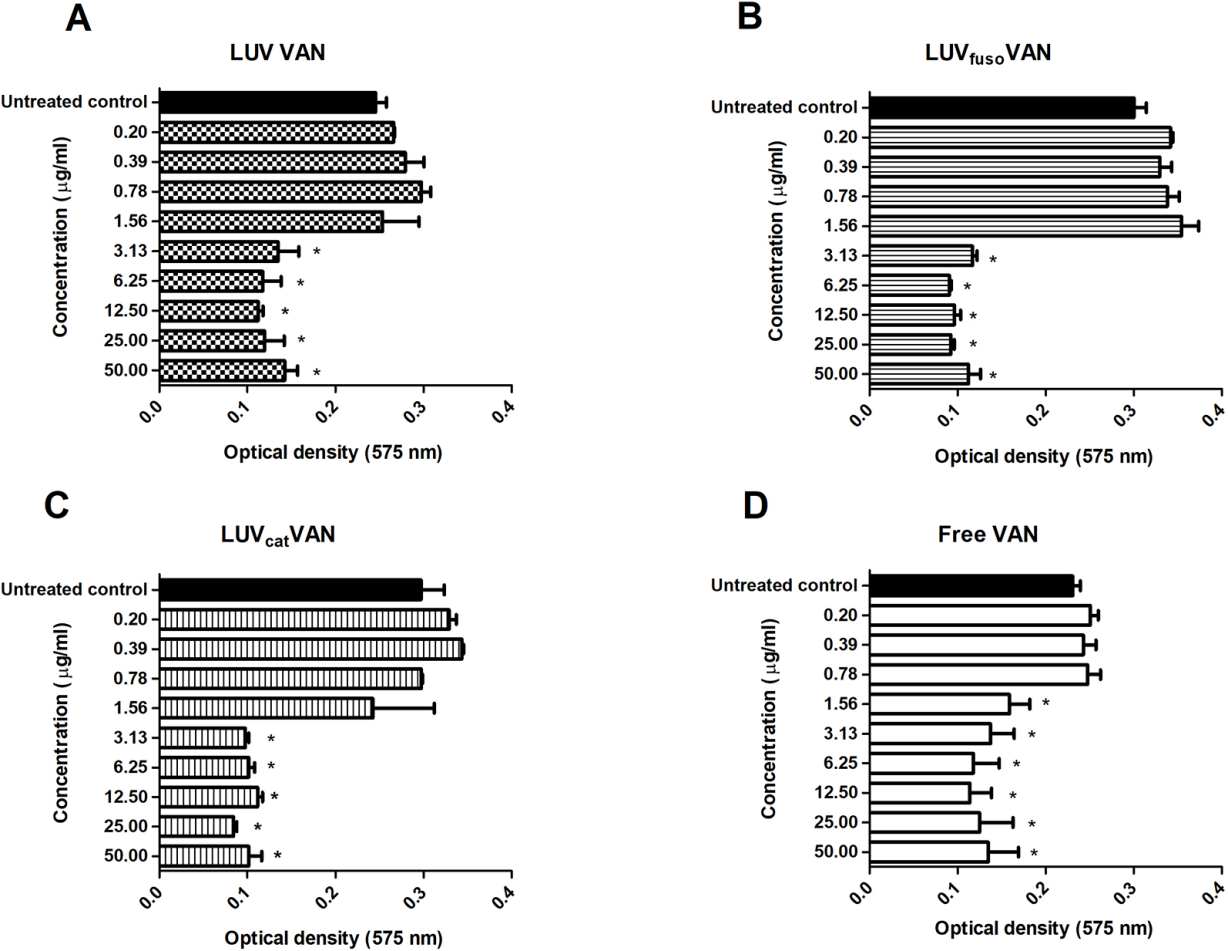
Free and liposome-encapsulated VAN formulations for their inhibitory effects on S*. aureus* ATCC 29213 biofilm adherence. Mean ( ± SD) optical density values of *S. aureus* biofilms treated with different concentrations of VAN encapsulated into LUV VAN **(A)**, LUV_fuso_ VAN **(B)**, LUV_cat_ VAN **(C)**, or free VAN solution **(D)**. The asterisk “*” indicates statistically significant difference between the drug treatment and its respective untreated control at *p* < 0.05 (One-way ANOVA, followed by Tukey’s post-hoc test).

Next, the formulations were tested for their inhibitory effects on S*. aureus* ATCC 29213 biofilm adherence and formation. As shown in **Figure 4**, treatment with all formulations inhibited biofilm formation in a dose-dependent fashion. Free VAN was found to inhibit biofilm formation at MIC (1.56 µg/ml) and higher concentrations as compared to the untreated biofilm control, while the inhibitory effects of liposome-encapsulated VAN were only seen from 2 × MIC (3.13 µg/ml).

These results corroborate those of the *in vitro* release kinetics assay (**Figure 3**), in which encapsulated VAN showed a late release profile as compared to free VAN. These can be attributed to the % amount of VAN encapsulated into the liposomes, so that just a fraction amount of VAN is available to immediately act. Thus, it is likely that a lower amount of VAN molecules was initially released from the liposomal formulations, thereby slowing up their overall antimicrobial effects.

**Figure 5:**
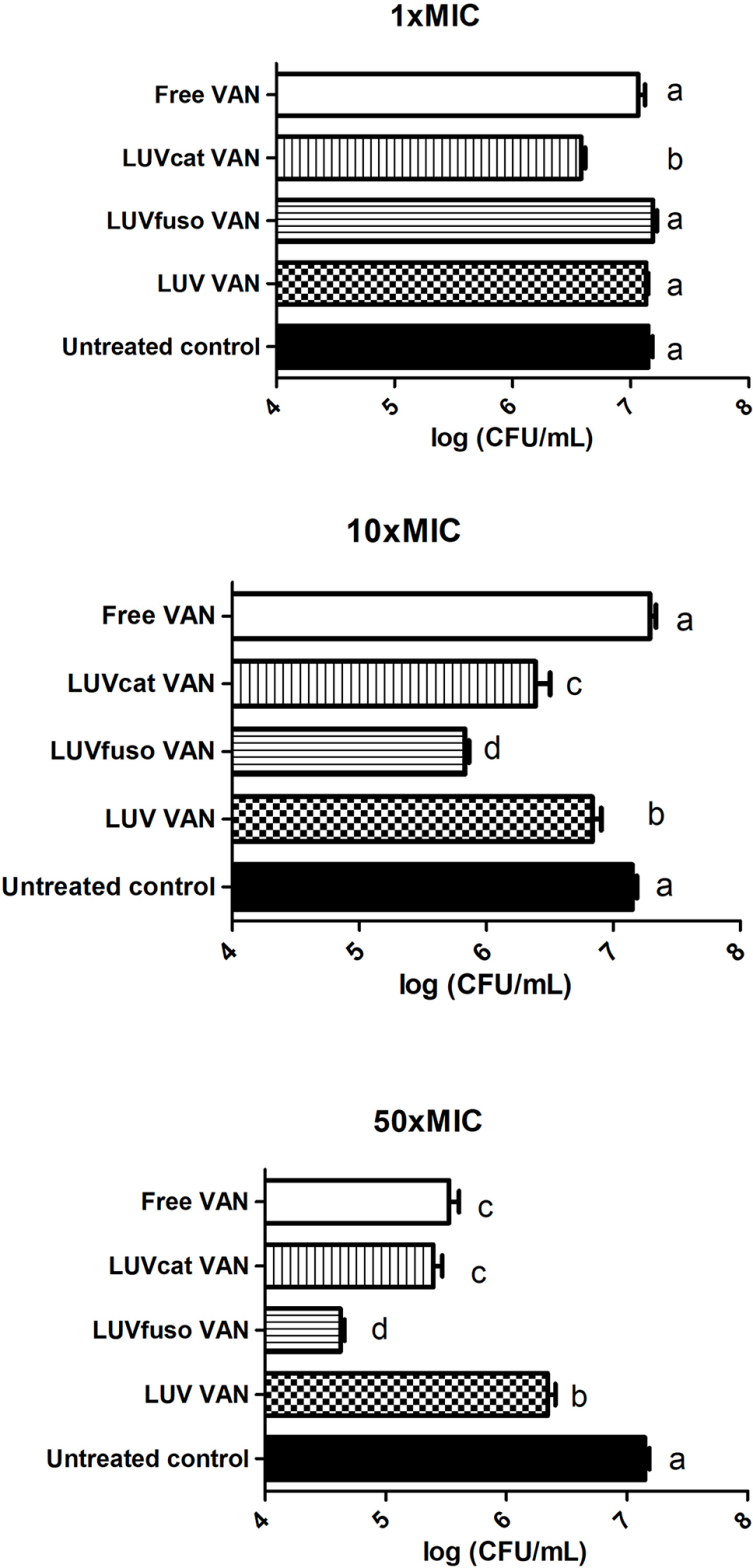
The inhibitory effects of the formulations on biofilm viability. Inhibitory effects of liposomal and plain formulations on *S. aureus* ATCC 29213 mature biofilm viability at 1 × MIC, 10 × MIC, and 50 × MIC. The values are expressed as mean ( ± SD) of CFU/ml. Different letters indicate significant differences between groups (*p* < 0.05 One-way ANOVA, with Tukey’s post-hoc test).

The inhibitory effects of the formulations on biofilm viability were also investigated. **Figure 5** shows the mean (± SD) CFU/ml (Log_10_) of biofilms treated for 24 h at 1 × MIC, 10 × MIC, and 50 × MIC. The data was compared among treatment groups and the untreated control. At 1 × MIC, only LUV_cat_ VAN caused a significant decrease in the number of viable biofilm cells (*p* < 0.01). Nevertheless, at 10 × MIC and 50 × MIC all formulations showed significant inhibitory effects as compared to the untreated control (*p* < 0.05). Free VAN was not able to affect biofilm viability significantly at 10 × MIC (p > 0.05), but it did at 50 × MIC (p < 0.05). When liposomal formulations were compared among themselves, we observed that LUV_fuso_ VAN had the most noticeable inhibitory potential on mature biofilms, followed by LUV_cat_ VAN and LUV VAN, with significant differences among them (p < 0.05).

The effects on mature biofilms treated with LUV_cat_ VAN and free VAN were found to be similar at 50 × MIC (p > 0.05) and greater than those promoted by LUV VAN (*p* < 0.05). LUV_fuso_ VAN was the most active formulation against *S. aureus* biofilm viability when compared to the other groups (*p* < 0.05). LUV_fuso_ VAN reduced biofilm viability by 3.5 Log_10_ CFU/ml (35×); LUV_cat_ VAN and free VAN caused a reduction of 2.5 Log_10_ CFU/ml (25×), while LUV VAN reduced biofilm viability by 1 log_10_ CFU/ml (10×) in comparison to the control.

## Discussion

Nosocomial and community-acquired MRSA infections remain a major concern in global health and have driven the adoption of public policies and medical research in this field (Honary et al., 2013; Elkhodairy et al., 2014; Holland et al., 2014). Evidence has shown the promising results of liposomal vesicles as drug carriers for pharmaceutical application (Kim et al., 1999; Nicolosi et al., 2010; Ma et al., 2011) (25, 26, 30). Herein, we report the development, characterization, and antimicrobial properties of experimental formulations containing VAN encapsulated into conventional, fusogenic and cationic liposomes. We compared the different formulations and demonstrated that the drug-delivery liposomes were more active than VAN in solution in reducing mature biofilm, with better efficacy for LUV_fuso_ VAN.

Our goal when selecting the liposomal formulations was to achieve greater interaction with bacterial cells and, thereby, facilitate penetration into mature biofilms. Conventional (LUV) liposomes contain a mixture of EPC and cholesterol, which increases the rigidity and stability of the vesicles (de Paula et al., 2012). LUV_fuso_ liposomes contain DOPE in their composition, which promotes destabilization of the lipid bilayer (towards inverse hexagonal structures) at acidic pH as it occurs in infected tissue. The use of DPPC was required for stabilization of the lipid bilayers due to the presence of DOPE. Additionally, EPC and CHEMS contribute to greater stability of the formulation (Aoki et al., 2015; Nicolosi et al., 2015). LUV_cat_ liposomes contained stearylamine, EPC, and cholesterol in their composition. Sa is a positively charged lipid that facilitates, through electrostatic interactions, adsorption in the negatively charged bacterial biofilm (Balazs and Godbey, 2011). In order to prevent lipid oxidation, the antioxidant alpha-tocopherol was added to all liposomal formulations (de Paula et al., 2012).

The effect of VAN encapsulation was observed in changes in the Zeta values and size distribution in comparison to controls (**Table 1**). Such changes may have occurred because encapsulated VAN has a tendency to be located in the aqueous core or adjacent lipid-water interface near the polar head groups (Bozzuto and Molinari, 2015). This molecular location of VAN in liposomes could contribute to the reduction of size distribution homogeneity and enhance electrostatic attraction among liposomes, as VAN is positively charged. Similar results concerning vesicle size and PDI were also found with tetraether lipid liposomes (Uhl et al., 2017). Moreover, after 180 days of storage (4°C) the structural properties of the liposomes were maintained after VAN encapsulation, presenting desirable size and monodisperse distribution, as required for a drug delivery system.

LUV VAN and LUV_cat_ VAN showed higher %EE (32.5% and 10.1%, respectively) than those already reported (2.0% and 5.0%, respectively) using equivalent (conventional, cationic) liposomes, but prepared by sonication and containing 20 mg/ml VAN (Kadry et al., 2004). On the other hand, Nicolosi et al. (2010) observed greater %EE (65.8%) for fusogenic liposomes (prepared by the reverse-phase evaporation method) as compared to our findings (11.4%) (Nicolosi et al., 2010). According to the authors, the preparation method and drug concentration in the liposomal suspension may have influenced the high upload (Muppidi et al., 2012).

In this study, no significant difference was observed in the release kinetics of VAN-containing LUV_fuso_ and LUV_cat_. Both formulations released 12% of VAN after 1 h, whereas LUV VAN released 2% and free VAN 33%. The differences in the drug controlled release profile among the liposomal formulations may be a result of their diverse %EE (Lankalapalli et al., 2015; Liu et al., 2015). Recently (Lankalapalli et al., 2015), evaluated the release kinetics of VAN from conventional liposomes EPC : Chol liposomes with VAN (10 mg/ml), prepared by the ethanol injection method. The authors observed similar results to those found in our study regarding VAN release from LUV VAN liposomes, and different results with regard to release of free VAN, which was about 42% after 22 h. This divergence may be related to the free VAN concentration used in the donor compartment, which was 100 mg/ml in the study by Lankalapalli et al. and 10 mg/ml in our study.

It is also known that VAN exerts antibacterial action by inhibiting the synthesis of cell wall peptidoglycans (Howden et al., 2010). This drug has a high affinity to the D-Ala-D-Ala residue from the peptidoglycan precursor, lipid II, thereby blocking the addition of final precursors by transglycosylation and transpeptidation, which ultimately interrupts cell wall formation. In *S. aureus*, peptidoglycan biosynthesis takes place in the cell division septum in a specific site of the cytoplasmic membrane (Howden et al., 2010). Thus, in order to promote its effects on the cell wall, VAN molecules should penetrate approximately 20 layers of peptidoglycan to reach the division septum and bind to the protein fraction (L-lysine-D-alanyl-D-alanine) of murein monomers used as a substrate for glycosyltransferases. Depending on the bacterial cell cycle phase, the division septum can be completely formed or under formation (Nicolosi et al., 2010). Hence, the distance between the cell wall and the plasma membrane is shorter at the early phases of bacterial growth, which might have contributed to the bactericidal effects of free VAN. However, when bacterial growth reaches a final stage, the division septum is completely formed. As a result, the distance between the cell wall and the plasma membrane is wider, which may hinder the action of free VAN. In this case scenario, it is believed that encapsulated VAN could more effectively penetrate the cell wall and reach the periplasmic space, therefore promoting its antibacterial effects (Sande et al., 2012; Nicolosi et al., 2015). Such increased penetration can explain the improved antibiofilm activity observed in our study for the liposomal formulations.

The MIC values of liposome-encapsulated VAN on *S. aureus* ATCC 29213 observed in our study are in agreement with those found by Kadry et al. (2004). These authors reported MIC values of 0.75 µg/ml and 1.50 µg/ml for cationic and conventional liposomes, respectively. Another study found that encapsulation of VAN into conventional liposomes reduced by 2 the MIC against MRSA strains as compared to free VAN (Sande et al., 2012). This liposomal formulation was composed of DSPC : DCP:Chol (7:2:1, mol%) containing VAN at 50 mg/ml, which was 5 times higher than the VAN concentration used in our study.

Our findings indicate that free VAN at MIC had better inhibitory effects on early stages of biofilm formation than had the liposomal formulations. The latter inhibited biofilm adherence only from 2 × MIC, probably due to the encapsulation of vancomycin into the liposomes, with less free drug available to interact with forming biofilm. On the other hand, the liposomal formulations showed improved antibacterial activity than free VAN against mature biofilms, particularly LUV_fuso_ VAN which was the most effective. Therefore, encapsulated VAN showed greater bactericidal effects on mature biofilms probably due to its increased ability to penetrate the peptidoglycan layers, whereas free VAN remained trapped in the cell wall.

Fusogenic liposomes have an increased potential to interact with extracellular matrix and cell wall due to their ability to merge with lipid membranes (Forier et al., 2014; Nicolosi et al., 2015). These vesicles are composed of lipids that promote destabilization of the lipid bilayers (Forier et al., 2014) and their fusion with the bacterial cell wall was previously proved through flow cytometry, lipid-mixing assay, electronic transmission microscopy and immunochemistry (Beaulac et al., 1998; Sachetelli et al., 2000; Forier et al., 2014; Wang et al., 2016). These vesicles can pass through the cell wall and deliver VAN into the periplasmic space, thereby making it easier for the drug to reach the division septum and block peptidoglycan biosynthesis (Howden et al., 2010; Sande et al., 2012; Nicolosi et al., 2015). Besides, cationic liposomes may have a higher affinity for negatively charged biofilms, which can decrease VAN delivery time into the infectious focus (Kim et al., 1999; Kadry et al., 2004). Accordingly, these liposomes probably release VAN in the vicinities of the bacterial cell wall due to the affinity with its negative charge, resulting in inhibition of cell wall biosynthesis.

There are other studies with VAN-loaded liposomal formulations (Ma et al., 2011; Barakat et al., 2014), but very few tested the ability in inhibit or eradicate *S. aureus* biofilm, which is a more resistant form of growth and much less sensitive to antibiotics. In the present study, we compared two formulations that are claimed to be effective against bacterial growth: fusogenic and cationic vesicles. Both formulations were effective in reducing mature biofilm, but with superiority to fusogenic vesicles.

To the best of the author´s knowledge, there are only two studies that encapsulate vancomycin into fusogenic liposomes (Nicolosi et al., 2010; Garcia et al., 2017), but none of them tested the activity against *S. aureus*. In addition, there are other non-fusogenic VAN-loaded liposomes that were tested against *S. aureus*, but very few aimed to test against biofilm (Ma et al., 2011; Barakat et al., 2014). Other drug delivery systems have also been proposed to improve drug delivery at sites of infection and to overcome antimicrobial resistance, such as injectable and biodegradable hydrogels (Zhao et al., 2017; Qu et al., 2018; Liang et al., 2019; Qu et al., 2019), polymeric nanoparticles (Lakshminarayanan et al., 2018), metal-based nanoparticles (Brown et al., 2012; Noronha et al., 2017), carbon-based nanoparticles (Zhao et al., 2017; Jiang et al., 2018), etc. Contributing to the development and comparison of antibiotics delivery systems, the present study showed that the liposomes here tested can reduce the formation and viability of mature biofilm, in a way superior to free vancomycin.

## Conclusion

We demonstrated the successful development, characterization and stability of LUV, LUV_fuso_ and LUV_cat_ encapsulated VAN formulations. Liposomes improved the antimicrobial activity of vancomycin against *S. aureus* biofilm, with better efficacy for fusogenic vesicles. Future studies are needed to validate this formulation as a candidate for *S. aureus* infection control.

## Data Availability Statement

All datasets generated for this study are included in the article/supplementary material.

## Author Contributions

AS: Conception and design of the project. Acquisition of data. Analysis and interpretation of data. Writing and revision of the manuscript. Approval of the final version of the manuscript. VC: Acquisition of data. Analysis and interpretation of data. Final approval of the version to be published. LR: Acquisition of data. Analysis and interpretation of data. Writing and revision of the manuscript. Approval of the final version of the manuscript. IF: Writing and revision of the manuscript. Approval of the final version of the manuscript. FG: Conception and design of the project. Analysis and interpretation of data. Approval of the final version. EP: Conception and design of the project. Analysis and interpretation of data. Writing and revision of the manuscript. Approval of the final version. MF-M: Conception and design of the project. Analysis and interpretation of data. Writing and revision of the manuscript. Approval of the final version. KC-M: Conception and design of the project. Coordination and execution of the experiments. Analysis and interpretation of data. Writing and revision of the manuscript. Approval of the final version of the manuscript.

## Conflict of Interest

The authors declare that the research was conducted in the absence of any commercial or financial relationships that could be construed as a potential conflict of interest.
